# Artificial Intelligence in the Diagnosis of Onychomycosis—Literature Review

**DOI:** 10.3390/jof10080534

**Published:** 2024-07-30

**Authors:** Barbara Bulińska, Magdalena Mazur-Milecka, Martyna Sławińska, Jacek Rumiński, Roman J. Nowicki

**Affiliations:** 1 Department of Dermatology, Venereology, and Allergology, Faculty of Medicine, Medical University of Gdańsk, 80-214 Gdańsk, Poland; 2 Department of Biomedical Engineering, Faculty of Electronics, Telecommunications and Computer Science, Gdańsk University of Technology, 80-233 Gdańsk, Poland

**Keywords:** artificial intelligence, deep learning, dermoscopy, onychomycosis, nail

## Abstract

Onychomycosis is a common fungal nail infection that is difficult to diagnose due to its similarity to other nail conditions. Accurate identification is essential for effective treatment. The current gold standard methods include microscopic examination with potassium hydroxide, fungal cultures, and Periodic acid-Schiff biopsy staining. These conventional techniques, however, suffer from high turnover times, variable sensitivity, reliance on human interpretation, and costs. This study examines the potential of integrating AI (artificial intelligence) with visualization tools like dermoscopy and microscopy to improve the accuracy and efficiency of onychomycosis diagnosis. AI algorithms can further improve the interpretation of these images. The review includes 14 studies from PubMed and IEEE databases published between 2010 and 2024, involving clinical and dermoscopic pictures, histopathology slides, and KOH microscopic images. Data extracted include study type, sample size, image assessment model, AI algorithms, test performance, and comparison with clinical diagnostics. Most studies show that AI models achieve an accuracy comparable to or better than clinicians, suggesting a promising role for AI in diagnosing onychomycosis. Nevertheless, the niche nature of the topic indicates a need for further research.

## 1. Introduction

Onychomycosis is a common fungal infection of the nail that affects a significant part of the population. The disease can be caused by a variety of fungal species, including dermatophytes, yeasts, and non-dermatophytes [1]. Fungal infections are typically diagnosed using microscopy analysis after exposing tissue scrapings to KOH solution (alternatively sodium sulfide, sodium hydroxide, Parker blue black ink, or calcofluor white), by microbiological fungal culture, or histologically by biopsy staining using Periodic acid–Schiff stain (PAS) or Grocott methenamine-silver. A nail scraping KOH microscopic analysis is dependent on the examiner’s subjective interpretation and varies highly with sensitivity, from 55.3% to 93.0% [2,3]. A microscopic examination provides limited information on the viability of fungal spores and hyphae. If antifungal treatment has already begun before the test, it can result in false negatives [4]. Culture is considered a gold standard for fungal identification. It can help determine the viability and species of the pathogen, but often requires 4–6 weeks or longer for definitive results due to the slow-growing nature of many fungi. The culture can be affected by environmental contaminants, though the use of antimicrobial solutions in the growth medium can help to inhibit bacterial development [5]. Examination using PAS staining usually requires performing nail clipping or, on infrequent occasions, biopsies [6]. Molecular techniques, such as PCR (Polymerase Chain Reaction) and MALDI-TOF MS (Matrix-Assisted Laser Desorption–Ionisation–Time of Flight Mass Spectrometry) are increasingly employed in routine fungal diagnostics due to their ability to swiftly detect fungi with high sensitivity, often within a few hours. However, these advanced methods have limitations; they cannot determine fungal viability and are relatively expensive, limiting access for practitioners dealing with suspected cases of onychomycosis [7].

Dermoscopy is a technique used to examine the skin and its adnexa in detail, with magnification up to 10 times. This enhances the identification of features that are not visible to the naked eye, aiding in the identification of skin conditions. The procedure has emerged as a valuable tool in onychomycosis diagnosis, offering a non-invasive, rapid, and precise method for evaluating nail abnormalities. Onychoscopy reveals distinct characteristics that facilitate the pattern of fungal nail destruction and differentiation from other nail disorders. These features include a jagged proximal margin, longitudinal striae with multicolor vertical streaks, and spiked patterns indicative of fungal invasion. Additionally, subungual hyperkeratosis, with a ruin-like appearance, is commonly observed [8]. In the study performed by Abu El-Hamd et al., among 52 patients suffering from onychomycosis, the most common dermoscopic features were cracking (92.8%), onycholysis (91.1%), discoloration (83.9%), thickening (71.4%), subungual hyperkeratosis (53.6%), paronychia (39.3%) and transverse ridges (14.2%). Less commonly observed were splinter hemorrhage (5.4%), pitting (3.6%), longitudinal ridges (3.6%), and thinning (1.8%) [9]. According to Yorulmaz and Yalcin, jagged proximal edges with spikes of the onycholytic area were the most common dermoscopic findings in 81 patients with fungal nail infection [10].

A significant feature of dermoscopic examination is its capacity to assist in differential diagnosis [11]. It can facilitate distinction between diseases such as melanoma, which can be mistaken for the more common and relatively benign melanonychia in the course of onychomycosis.

Artificial intelligence has made advancements in medicine, utilizing machine learning algorithms, natural language processing, and other AI technologies to analyze complex medical data, enhance diagnostic accuracy, personalize treatment plans, and improve patient outcomes [12]. In radiology, it is used to analyze medical images, such as X-rays, CT scans, and MRIs [13,14] to detect abnormalities and assist in the diagnosis of conditions like cancer [15] and cardiovascular diseases [16]. Another example is pathology, where AI aids in the examination of tissue samples, enhancing the accuracy and efficiency of identifying diseases [17].

In dermatology, AI has shown great promise in diagnosing skin conditions by analyzing medical images. AI algorithms have been developed to evaluate images of the skin to detect and classify conditions such as melanoma [18], psoriasis [19], and acne [20]. These algorithms can enhance the diagnostic capabilities of dermatologists by providing a second opinion or flagging potential issues that may not be immediately apparent to the human eye [21]. The integration of AI into dermatology not only improves diagnostic accuracy but also increases efficiency by reducing the time required to analyze images. In the same way, artificial intelligence has the potential to improve the diagnosis of onychomycosis.

The accurate diagnosis of onychomycosis is crucial for the effective treatment and management of the disease. Misdiagnosis can lead to inappropriate treatments, prolonged infection, and unnecessary side effects. Accurate identification ensures that patients receive the correct antifungal therapy, improving outcomes and reducing the risk of complications. In this literature review, we aim to provide an overview of the current research on the use of artificial intelligence in the diagnosis of onychomycosis. With the ability to process large amounts of data and identify complex patterns, AI has the potential to significantly improve the accuracy and efficiency of diagnosing this common and often challenging condition.

## 2. Materials and Methods

### 2.1. Study Design

This systematic review was conducted based on the Preferred Reporting Items for Systematic Reviews and Meta-Analyses (PRISMA) guidelines.

### 2.2. Eligibility Criteria

**Figure 1:**
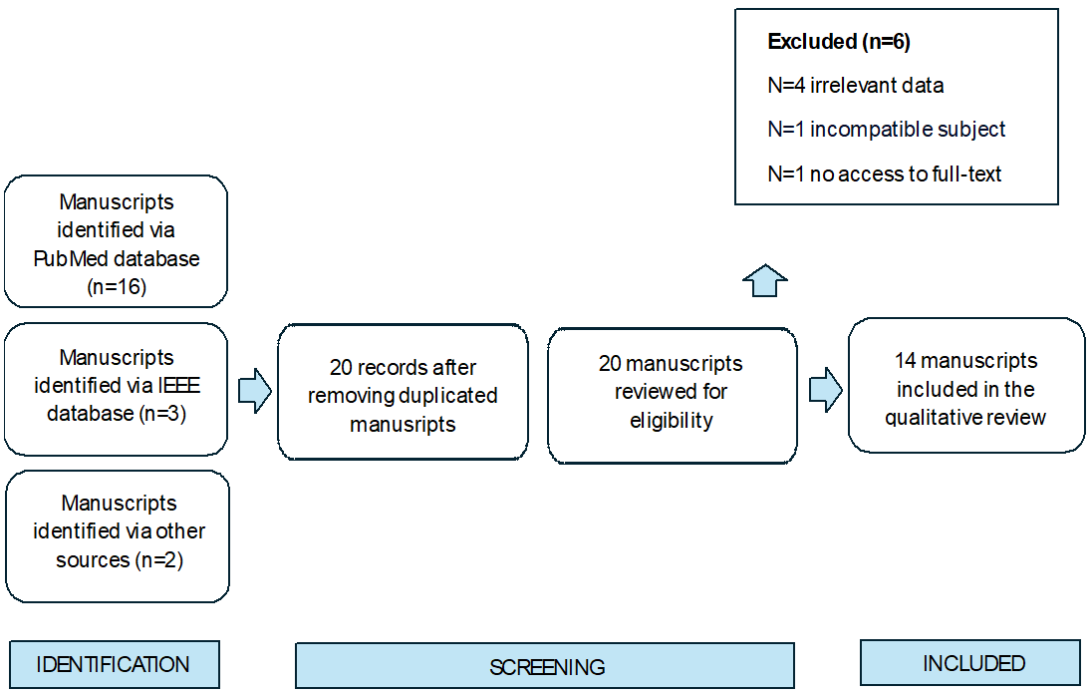
PRISMA 2020 diagram for the selection process of studies for a systematic review of AI-augmented diagnosis of onychomycosis up to June 2024.

The manuscripts included topics of artificial intelligence tools for the diagnosis of nail fungal infections. During the screening process, we did not exclude any study design, which resulted in the inclusion of literature reviews, peer-reviewed articles, and conference reports. The publications were restricted to the years 2010-2024, and the studies included humans with no specified age, sex, or language limitations. The selection process was based on PRISMA 2020 criteria, and is presented in Figure 1.

### 2.3. Information Sources, Search, and Study Selection

Literature retrieval was conducted from May to June 2024 in the PubMed and IEEE databases. The investigation strategy was adapted to the Medical Subject Headings (MeSH) phrases and introduced to the search engines of the Pubmed database as follows: (“onychomycosis”[MeSH] OR onychomycos*[tiab] OR “tinea unguium”[tiab]) AND (“diagnosis”[MeSH] OR “diagnosis”[Subheading] OR diagnos*[tiab] OR detect*[tiab] OR confirmation*[tiab]) AND (“artificial intelligence”[MeSH] OR “artificial intelligence*”[tiab] OR ai[tiab] OR “machine learning*”[tiab] OR “neural network*”[tiab] OR “deep learning*”[tiab]). MeSH phrases were also applied to the IEEE database. The EndNote (Clarivate Analytics) program was utilized to merge the search results and automatically remove duplicates. Two independent reviewers (B.B. and M.M.-M.) first screened the abstracts of identified manuscripts. Next, the full texts of potentially adequate manuscripts were reviewed. Any disagreements were resolved by reaching a consensus.

### 2.4. Data Collection and Quality Assessment

The retrieved articles were attentively reviewed by both B.B. and M.M.-M., and, when available, the following data were extracted: the name of the first author, the year of publication, the study type, the number of samples/patients, the clinical diagnosis, the model of the sample (image) assessment, the model of AI described, the performance of the AI, and a comparison of diagnostic ability to dermatologists/medical professionals. Due to the niche and still developing nature of the topic, as well as the diversity of the studies, a meta-analysis was not conducted. B.B. and M.M.-M. applied the Quality Assessment of Diagnostic Accuracy Studies-2 (QUADAS-2) checklist to exclude research studies with a potentially high risk of bias [22]. Table 1 presents the domains considered: patient selection, index test, reference standard, flow and timing. All the studies underwent a quality assessment by B.B. and M.M.-M.

## 3. Results

A total of 14 manuscripts were selected for inclusion in the literature review on the AI-augmented diagnosis of onychomycosis. These studies were published between 2017 and 2023, and the research centers of the scientists conducting these studies were primarily based in South Korea, China, the USA, and Germany. Table 2 presents the following information: manuscript reference, study type, number of samples/patients, clinical diagnosis, model of the sample (image) assessment, AI model described, AI performance, and comparison of diagnostic ability to dermatologists/medical professionals.

Three manuscripts were literature reviews/mini-reviews, and eleven were research studies (prospective, retrospective, or combined). Ten studies included clinical or dermoscopic images, two involved histopathology slides, one presented microscopic images with KOH examination, and one considered all three types of onychomycosis images. Four studies focused on more than just onychomycosis detection, including images of other nail, skin, mucous membranes, and hair lesions such as psoriasis, atopic dermatitis, lupus erythematosus, bullous pemphigoid, tinea pedis and corporis.

### 3.1. Clinical and Dermoscopic Images

Most studies reviewed utilized clinical images of onychomycosis for the model design. Among these, Schielein et al. demonstrated the best results. The performance exceeded other networks, with the highest performance reaching a sensitivity, specificity, and accuracy of 100% for 270 onychomycosis images. The best accuracy was achieved using the CNNs InceptionV3, Xception, and ResNet50 between all the algorithms assessed by Schielein et al. [28]. It should be noted, however, that the classification was binary, meaning that the model was trained to distinguish between onychomycosis and a healthy nail only. Furthermore, the images were reviewed by two dermatologists before training and all incongruous photos were excluded. The exclusion criteria were: uncertainty about the accuracy of the provided diagnosis, the presence of other skin conditions in the image, and the widespread use of colored antiseptic agents.

The second most commonly described imaging model utilized dermatoscopy. In these cases, AI performed best in the model presented by Zhu Xianzhong et al. In the study, 603 images of onychomycosis were applied to train the CNN model. Statistically significant onychomycosis characteristics included subungual keratosis, longitudinal striae, jagged edge, distal irregular termination, marble-like turbid area, and cone-shaped keratosis. Three Faster R-CNN models were trained for binary detection: normal vs. nail disorder (dataset A1), onychomycosis vs. other nail disorder (dataset A2), and dermoscopic pattern detection (dataset A3). An ensemble model was then created using two classes (normal nail or nail disorder) and another two classes (onychomycosis or not). The outputs of the three models were combined. The sensitivity, specificity, and accuracy of the model were 78.5%, 93.0%, and 87.5%, respectively, which exceeded the results from 54 dermatologists (the mean sensitivity, specificity, and Youden index for attending doctors and residents were 64.3% ± 22.0%, 78.4 ± 12.9%, and 0.427 ± 0.188, respectively; for senior doctors and associate seniors, the values were 67.8 ± 22.4%, 76.6 ± 13.0%, and 0.444 ± 0.172, respectively) [24].

### 3.2. Histopathology Slides of Nail Clippings

A lower percentage of the reviewed studies included pathomorphological slides of nails affected by onychomycosis. Jansen et al. trained a segmentation model and subsequently generalized it into an algorithm capable of distinguishing samples as positive (tinea) or negative (no tinea). The performance of the AI tool was then compared against 11 board-certified dermatopathologists using 74 validated slides. The U-Net architecture achieved a sensitivity of 94.0%, a specificity of 77.0%, a positive predictive value (PPV) of 88.0%, and a negative predictive value (NPV) of 87.0%. Meanwhile, the accuracy of the dermatopathologists varied between 71.4% and 97.3% [23].

Decroos et al. used a convolutional neural network (CNN) similar to VGG-13, consisting of 13 convolution layers with extended dilation, allowing the kernel size to be enlarged without increasing the number of parameters needed. The analyzed pathomorphological slide was split into “patches” (small images), and the probability of onychomycosis for each patch was estimated. By considering the 10 patches with the highest onychomycosis probabilities, CNN applied a threshold to obtain a binary decision (tinea or no tinea). The performance of this AI model was compared to four dermatopathologists. For two specialists with significant practical experience, their performance was slightly better than the algorithm. However, the opposite was observed when the AI was compared to dermatopathologists who had recently completed dermatopathology training. The PPVs (Positive Predictive Values), with corresponding two-sided 95.0% logit confidence intervals among the four dermatopathologists, varied between 88.9% and 99.0%, while the AI with dichotomized PPV was 97.9% [6].

### 3.3. Microscopic Images of Nails with KOH Examination

In the review, research involving microscopic images of nails examined with KOH was focused on the least. Yilmaz et al. based their study on 160 full-field microscopic photographs of nails affected by onychomycosis and 297 full-field microscopic photographs of dissolved keratin obtained from normal nails. The models VGG16 and InceptionV3 were developed using these data, resulting in mean sensitivity rates of 75.0 ± 2.7% and 74.9 ± 4.5%, specificity rates of 92.7 ± 1.2% and 93.8 ± 1.7%, and accuracy rates of 88.1 ± 0.8% and 88.8 ± 0.4%, respectively. The performance of these models was compared to that of 16 dermatologists, whose mean sensitivity was 74.8 ± 19.5%, mean specificity was 74.3 ± 18.0%, and mean accuracy was 74.5 ± 8.6%. The AI models demonstrated superior accuracy and specificity compared to dermatologists, but were less sensitive [25].

## 4. Discussion

The application of artificial intelligence (AI) in medical diagnostics has gained significant attention in recent years. This systematic review presents the use of AI-augmented diagnosis of onychomycosis using clinical pictures, dermoscopy, histopathological slides and KOH-treated microscopic images.

### 4.1. Dermoscopic Images

**Figure 2:**
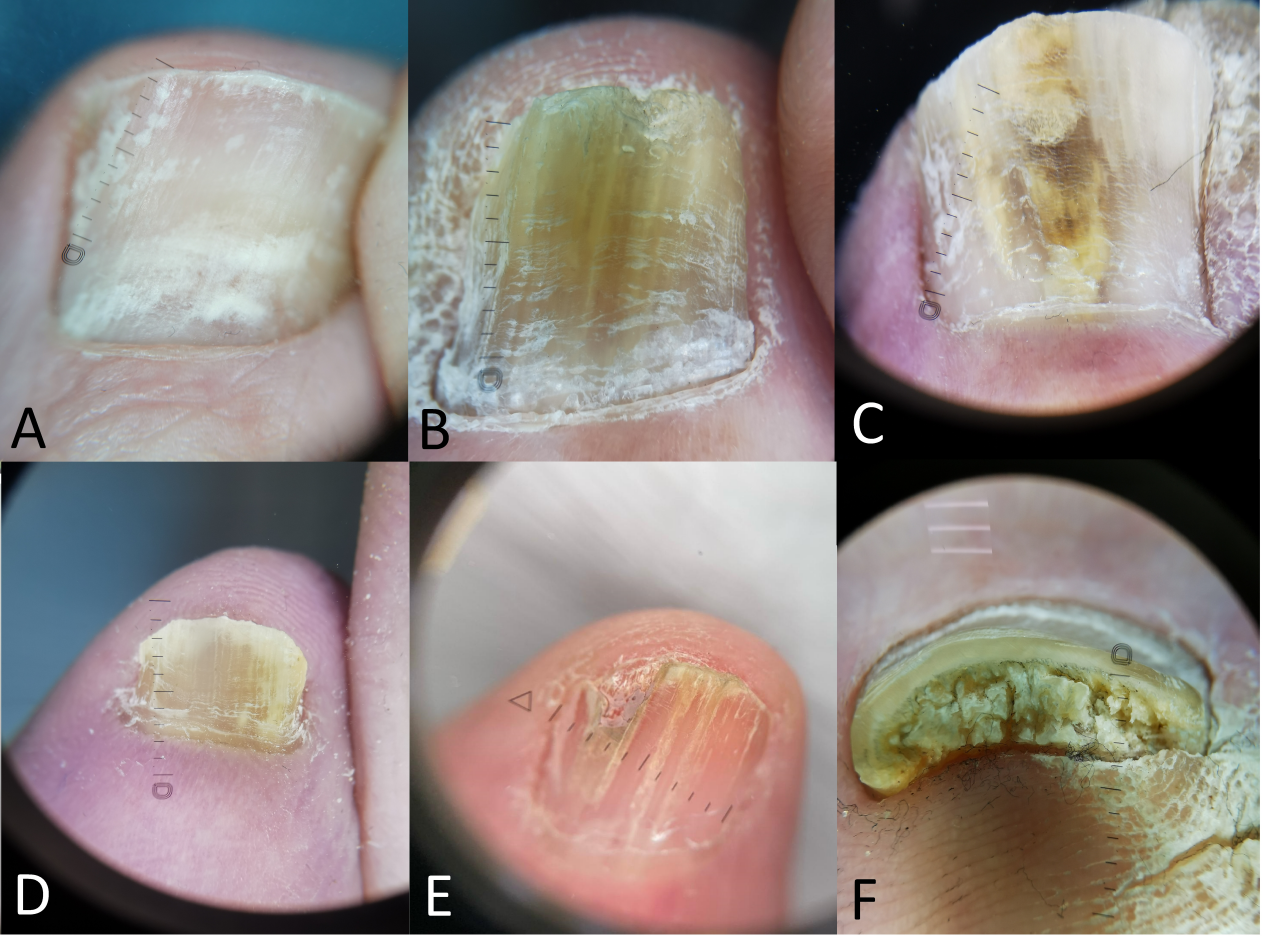
Dermoscopic images of onychomycosis features: (**A**) leukonychia (white fluffy shadow), (**B**) longitudinal striae with multicolor streaks, (**C**) chromonychia (brown, yellow and white colors), (**D**) longitudinal white striae, (**E**) jagged distal edge, (**F**) subungual hyperkeratosis.

Dermoscopy has emerged as a valuable tool in the diagnosis of onychomycosis, offering a non-invasive, rapid, and precise method for evaluating nail abnormalities. It reveals distinct characteristics of onychomycosis, such as longitudinal striae, spiked patterns, and a jagged proximal edge, which are indicative of fungal invasion (Figure 2). Moreover, it enhances the visualization of subtle changes in the nail plate, such as homogenous opacity and irregular borders, providing critical insights that support clinical diagnosis and guide treatment decisions.

Various AI models have been developed to interpret dermatoscopic images, leveraging the unique advantages of this technique in diagnosing nail disorders [29]. These models often use standard and widely used CNN classifiers like VGG and ResNet [27,28,29], or detectors like R-CNN [24], achieving accuracy, sensitivity, or specificity rates of over 90.0%. All of this is accomplished with images obtained quickly and non-invasively.

Integrating dermoscopy into routine clinical practice not only improves diagnostic accuracy and patient outcomes but also has significant implications for AI development. The systematic use of dermoscopy and the routine recording of dermatoscopic images can lead to the creation of larger and more diverse databases. This is particularly important for accumulating data on rare cases, which can be used to further refine and enhance AI models. As AI continues to evolve, these enriched datasets will be crucial in improving the models’ accuracy and reliability, ultimately leading to better diagnostic tools and enhanced patient care.

### 4.2. Histopathology Slides of Nail Clippings

Due to high specificity, relatively low costs, and being able to obtain the diagnosis quickly, pathomorphological examination is widely used. However, the tool does not remain without limitations. PAS-stained slide detection depends on the fungi load of the nail affected, but also on the laboratory preparation of the specimen. Jansen et al. underline the impact of possible tissue fragmentation and mention staining artifacts (such as bacteria or serum) as a reason for false tinea recognition by the algorithm [23]. Both Jansen et al. and Decroos et al. bring up an element of difficulty, namely the scan being out of focus [6,23]. This impairs the capability of both human and deep-learning models for proper diagnosis. Jansen et al. suggest that a higher number of scans could improve the overall performance [23]. It may be important to state that in the study conducted by Decroos et al., the AI learned to diagnose cases autonomously, while in the study carried out by Jansen et al., AI assisted the pathologists [6,23].

### 4.3. Images of Onychomycosis with KOH Examination

Despite the limited number of studies on the use of images of nails with KOH examination in AI-augmented diagnosis of onychomycosis, the results are promising. The average accuracy of this standard microscopic method is about 61%, whereas the study conducted by Yilmaz et al. reported an accuracy of 88.8% using the VGG16 model [25]. However, this type of convolutional neural network-based onychomycosis classification technique has its drawbacks. The method requires specific equipment and reagents, and can be time-consuming. The performance of microscopic examinations depends on subjective variables and heavily relies on the experience of the performer, potentially leading to false-negative results. Individuals with years of training and extensive sample viewing have a higher success rate compared to less trained investigators. Another issue when applying images to an AI algorithm is image quality, which can be affected by different backgrounds and lighting conditions. Yilmaz et al. suggest that using greyscale images can help mitigate this concern [25].

### 4.4. AI-Augmented Diagnosis of Onychomycosis

Machine learning algorithms, particularly convolutional neural networks (CNNs), have demonstrated high accuracy in diagnosing onychomycosis [33]. The systems have the capacity for continuous learning and improvement. This learning process ensures that AI tools remain up-to-date with the latest advancements in medical knowledge and diagnostic techniques, thereby maintaining their efficacy over time. The use of AI in diagnosis can lead to cost savings by reducing the need for multiple diagnostic tests and consultations. An early and accurate diagnosis, facilitated by AI, can prevent the progression of onychomycosis, reducing the need for more extensive and costly treatments. AI-powered decision support systems can assist clinicians, particularly those with less experience, in making reliable diagnoses. These systems can help bridge the gap between novice clinicians and seasoned experts, ensuring a more consistent quality of care. This is particularly relevant in the diagnosis of onychomycosis, where subtle differences in nail presentation can lead to misdiagnosis. AI algorithms can process and analyze images in a matter of seconds or minutes, providing rapid diagnostic results compared to traditional mycological diagnostic methods, such as fungal culture, which can take weeks for the results, during which time patients remain untreated. Rapid AI-driven diagnostics can lead to the timely initiation of appropriate treatments, improving patient outcomes. Mobile applications equipped with AI algorithms enable individuals to capture and upload nail images for remote diagnosis, making healthcare more accessible, especially in underserved areas. This can help to screen large populations, which is beneficial in regions with limited access to dermatologists.

The integration of machine learning (ML) and artificial intelligence in the diagnosis of onychomycosis, a common fungal nail infection, has shown promising potential. However, this technological advancement also comes with notable challenges. The effectiveness of AI algorithms heavily depends on the quality and diversity of the training data. If the dataset is biased or lacks representation from diverse populations, the AI system may produce inaccurate results. Ensuring that datasets are comprehensive and representative is crucial to avoid biases and improve the generalizability of the AI model. As CNNs function by classifying the content of an image, there is a possibility for misclassifications if an image does not fit any given category. Additionally, AI is as yet unable to differentiate between fungal species, necessitating fungal culture for species identification. The integration of AI in medical diagnostics raises several regulatory and ethical issues. Ensuring compliance with healthcare regulations and standards is essential to guarantee patient safety and data privacy. Ethical considerations related to AI use, such as consent, accountability, and potential biases, must also be addressed. Robust regulatory frameworks and ethical guidelines are needed to effectively navigate these challenges. A survey by Polesie S. et al. on 1271 dermatologists revealed that while most clinicians have a positive view on AI as a diagnostic and therapeutic tool, a small group expressed concern about the potential of being replaced by machine learning [36]. This indicates a possible issue of acceptance within the medical community. A proper understanding of AI terminology, methods, and outcome data is crucial to alleviate concerns and foster acceptance among healthcare professionals. There is a risk of over-reliance on AI tools, which might lead clinicians to neglect their clinical skills and judgment. Striking a balance between leveraging AI for diagnostic support and maintaining the critical thinking and expertise of medical professionals is essential to ensure high-quality patient care.

## 5. Conclusions

Artificial intelligence offers significant advantages in the diagnosis of onychomycosis, including enhanced accuracy, efficiency, continuous improvement, and decision support for clinicians. However, challenges such as data quality and bias, interpretability, regulatory and ethical concerns, as well as acceptance by the medical community should be carefully addressed. 

## Figures and Tables

**Table 1:** Evaluation of the risk of bias and applicability of primary diagnostic accuracy in reviewed studies.

Study	Risk of Bias				Applicability Concerns		
	Patient Selection	Index Test	Reference Standard	Flow and Timing	Patient Selection	Index Test	Reference Standard
Jansen, J Fungi (Basel), 2022 [23]	Moderate	Moderate	Low	Moderate	Moderate	Low	Low
Zhu, Mycoses, 2022 [24]	Low	Low	Low	Low	Low	Low	Low
Yilmaz, Mycoses, 2022 [25]	Low	Low	Low	Low	Low	Low	Low
Decroos, Acta Derm Venereol, 2021 [6]	Low	Low	Low	Low	Low	Low	Low
Nigat, J Healthc Eng, 2023 [26]	Moderate	Moderate	Moderate	Moderate	Moderate	Low	Moderate
Han, PLOS ONE, 2018 [27]	Low	Low	Low	Low	Low	Low	Low
Schielein, J Eur Acad Dermatol Venereol, 2023 [28]	Moderate	Moderate	Moderate	Moderate	Moderate	Moderate	Low
Kim, PLOS ONE, 2020 [29]	Low	Low	Low	Low	Low	Low	Low
Nijhawan, Conference SITIS, 2017 [30]	Moderate	Moderate	Moderate	Moderate	Moderate	Low	Moderate
Düzayak, SAUJS, 2023 [31]	Moderate	Moderate	Moderate	Moderate	Moderate	Moderate	Moderate
Marulkar, Conference CONIT, 2023 [32]	Moderate	Moderate	Moderate	Moderate	Moderate	Low	Moderate

**Table 2:** Artificial intelligence and onychomycosis. Study characteristics and AI descriptions summarized (N = 14).

First Author, Journal, Year	Study Type	Number of Samples	Diagnosis	Model of Picture Assessment	Model of AI Described	Performance of the Test	Comparison of Diagnostic Ability to Dermatologists/Medical Professionals
Gupta, J Cosmet Dermatol, 2022 [33]	Review	N/A	Onycho- mycosis	Clinical pictures of nails	CNN	N/A	Comparable/Superior
Gupta, J Fungi (Basel), 2022 [34]	Review	N/A	Onycho- mycosis	Clinical pictures of nails, Histopathology slides of PAS-stained nail clippings, Greyscale microscopic images of nail with KOH examination	CNN	N/A	Comparable/Superior
Jansen, J Fungi (Basel), 2022 [23]	Prospe- ctive study	664 histo- pathology slides	Onycho- mycosis	PAS-stained nail clipping slides manually annotated and used to train a U-NET model for binary segmentation	U-NET-based segmentation approach	Sensitivity/specificity/ accuracy 94.0%/77.0%/86.5%, respectively	Comparable.
Zhu, Mycoses, 2022 [24]	Prospe- ctive study	603 pictures of nails	Onycho- mycosis	Training datasets consisted of dermoscopic pictures of onychomycosis, nail psoriasis, traumatic onychodystrophy, and normal nails	Faster region-based convolutional neural networks	Sensitivity/specificity/ accuracy: 78.5%/93.0%/87.5%, respectively	Superior
Yilmaz, Mycoses, 2022 [25]	Prospe- ctive study	160 microscopic images of nails	Onycho- mycosis	Training datasets consisted of microscopic photographs of nails containing fungal structures and of dissolved keratin from normal nails	VGG16 and InceptionV3 models	For the VGG16 model, the InceptionV3 model and dermatologists, mean sensitivity: 75.0 ± 2.7%, 74.9 ± 4.5% and 74.8 ± 19.5%, mean specificity rates were 92.7 ± 1.2%, 93.8 ± 1.7% and 74.3 ± 18.0%, respectively, and mean accuracy rates were 88.1 ± 0.8%, 88.8 ± 0.4% and 74.5 ± 8.6%, respectively	Comparable/Superior
Lim, Dermatol Pract Concept, 2023 [35]	Mini Review	N/A	Onycho- mycosis	Clinical nail pictures	Two-layered feedforward neural networks computing the combined output of ResNet-152 and VGG-19	Sensitivity/specificity/area under the curve: (96.0/94.7/0.98), (82.7/96.7/0.95), (92.3/79.3/0.93), and (87.7/69.3/0.82) for the B1, B2, C, and D datasets, respectively	Superior
Decroos, Acta Derm Venereol, 2021 [6]	Retro- spective study	199 histo- patho- logy slides	Onycho- mycosis	PAS-stained onychomycosis slides manually annotated and utilized to train the AI model	CNN architecture similar to VGG-13 (26), but introduces dilation to the convolution operations	Sensitivity/specificity/area under the curve: 94.1%/98.0%/0.9601	Comparable
Nigat, J Healthc Eng, 2023 [26]	Prospective study	Clinical pictures of: Tinea unguium/ Onychomycosis 120, Tinea capitis 120, Tinea pedis 96, Tinea corporis 71	Tinea unguium/ Onychomycosis, Tinea capitis, Tinea pedis, Tinea corporis	Clinical pictures of lesions	HSFDC CNN model	Sensitivity/specificity/ accuracy/precision/F1 score: 86.4%/95.4%/93.3% /87.3%/86.8%, respectively	N/A
Han, PLOS ONE, 2018 [27]	Retro- spe- ctive study	Clinical pictures of 6673 nails	Onycho- mycosis	Training datasets consisted of linical images of onychomycosis, nail dystrophy, onycholysis, melanonychia, normal, nails, and others (subungual hemorrhage, paronychia, subungual fibroma, ingrown nail, pincer nail, periungual wart, etc.)	ResNet-152 + VGG-19 + feedforward neural networks models	Sensitivity/specificity/area under the curve: B1 dataset 96.0%/94.7%/0.98, B2 dataset 82.7%/96.7%/0.95, C dataset 92.3%/79.3%/0.93, D dataset 87.7%/69.3%/0.82, respectively	Superior
Schielein, J Eur Acad Dermatol Venereol, 2023 [28]	Retro- spe- ctive study	Clinical images: 276 onychomycosis, 1200 psoriasis, 1038 atopic dermatitis, 726 lupus erythematosus, 881 bullous pemphigoid	Onycho- mycosis, Psoriasis, Atopic dermatitis, Lupus erythematosus, Bullous pemphigoid	During AI training, the ‘normality’ category included images of the pathology, while the ‘outlier’ category had images from the other four pathologies	CNNs models: VGG-16, VGG-19, Inceptionv3, Xception, ResNet50	Among all networks the highest performances for onychomycosis pictures reached: Sensitivity/ specificity/accuracy 100%/100%/100%, respectively	N/A
Kim, PLOS ONE, 2020 [29]	Prospe- ctive study	57 pictures of nails	Onycho- mycosis	Training datasets consisted of clinical and dermoscopic pictures of onychomycosis and nail dystrophy	ResNet-152 and VGG-19, RCNN (backbone network = VGG-16) models	Sensitivity/specificity/area under the curve 70.2%/72.7%/0.751, respectively	Comparable
Nijha- wan, Conference SITIS, 2017 [30]	Confe- rence report	4190 pictures (including 482 of onychomycosis)	Onycho- mycosis, Beau’s Lines, hyperpigmentation, koilonychia, leukonychia, psoriasis, onychorrexis, paronychia, pincer nails, subungulal hematoma, yellow nail syndrome	Training datasets consisted of clinical nails pictures of onychomycosis, subungual hematoma, Beau’s lines, yellow nail syndrome, psoriasis, hyperpigmentation, koilonychias, paroncychia, pincer nails, leukonychia, onychorrhexis	A CNN with RELU (“non-saturating nonlinearity”)	In Scenario 3 *, sensitivity/specificity /accuracy were the highest (a total of four scenarios considered for accuracy assessment), at 0.91/0.88/84.6%, respectively; * the image was split into two parts again, and supplied the upper and lower halves to different CNNs, which also supplyied the full image to another CNN, combining the three feature vectors to form one, on which the final classification was applied using RF	Comparable
Düzayak, SAUJS, 2023 [31]	Prospe-ctive study	242 pictures of nails	Onycho-mycosis	Training datasets consisted of clinical nails pictures of onychomycosis and other nail conditions (not specified)	ANN (artificial neural networks), SVM (Support Vector Machine), EDT (Ensemble Decision Trees)	Proposed model Sensitivity/specificity /accuracy: 0.90/0.89/89.7%, respectively	N/A
Marulkar, Confe- rence CONIT, 2023 [32]	Confe- rence report	18,025 pictures of nails	Nail diseases categorized by features (including onychomycosis)	Training datasets consisted of 9 nail picture classes based on the clinical disease features	RF, KNN and CNN with SVM	The greatest achieved accuracy of 87.3% suggested approach’s sensitivity/specificity was 0.91/0.88, respectively	Comparable

## Data Availability

Not applicable.
